# Overexpression of *SmSCR1* Promotes Tanshinone Accumulation and Hairy Root Growth in *Salvia miltiorrhiza*

**DOI:** 10.3389/fpls.2022.860033

**Published:** 2022-03-08

**Authors:** Wei Zhou, Shuai Wang, Yafang Shen, Yunhui Liu, Itay Maoz, Xiankui Gao, Chengan Chen, Tingyao Liu, Can Wang, Guoyin Kai

**Affiliations:** ^1^Laboratory for Core Technology of Traditional Chinese Medicine (TCM) Quality Improvement and Transformation, School of Pharmaceutical Sciences, The Third Affiliated Hospital, School of Pharmacy and Academy of Chinese Medical Science, Zhejiang Chinese Medical University, Hangzhou, China; ^2^Department of Postharvest Science, Agricultural Research Organization, Volcani Center, Rishon LeZion, Israel

**Keywords:** *Salvia miltiorrhiza*, GRAS transcription factor, tanshinone accumulation, hairy root growth, regulatory mechanism

## Abstract

Lipid-soluble tanshinone is one of the main bioactive substances in the medicinal plant *Salvia miltiorrhiza*, and its medicinal demand is growing rapidly. Yeast extract (YE) modulates the tanshinone biosynthesis, but the underlying regulatory network remains obscure. In this study, a YE-responsive transcriptional factor Scarecrow1 (*SCR1*) was identified in *S. miltiorrhiza* from the YE-induced transcriptome dataset. *SmSCR1* is located in the nucleus. Overexpression of *SmSCR1* in *S. miltiorrhiza* roots resulted in a significantly higher accumulation of tanshinone than the control, with the highest 1.49-fold increase. We also detected upregulation of tanshinone biosynthetic genes, *SmSCR1* and *SmHMGR1*, and distinct alteration of growth and development of the hairy roots in the overexpression lines compared to the control. An inverse phenotype was observed in *SmSCR1*-SRDX suppression expression lines. We found that *SmSCR1* can bind to the promoter of *SmCPS1* to induce its expression. This study provides new insight into the regulatory mechanism on the growth and development of hairy roots, tanshinone accumulation, and the metabolic engineering of bioactive compounds in *S. miltiorrhiza*.

## Introduction

*Salvia miltiorrhiza* Bunge is a Chinese herbal medicine that belongs to the *Salvia* genus in the Lamiaceae family (Jia et al., 2019; Jung et al., 2020). In Asian countries, dried roots and stems of *S. miltiorrhiza* are a common treatment for cardiovascular system-related diseases (Hao et al., 2015). The bioactivity of *S. miltiorrhiza* is associated with lipid-soluble tanshinones, including tanshinone I (TA-I), cryptotanshinone (CT), dihydrotanshinone (DT), and tanshinone IIA (TA-II) (Fu et al., 2020). Increasing attention has been given to the abovementioned compounds in recent years, mainly focusing on improving the yield of tanshinone in *S. miltiorrhiza*.

Tanshinones are a type of diterpenoids produced in two phases in *S. miltiorrhiza* roots (Supplementary Figure 1). First, common terpenoid precursors [e.g., isopentenyl diphosphate (IPP) and dimethylallyl diphosphate (DMAPP)] are generated by two distinct processes (e.g., mevalonate (MVA) pathway in cytosol and 2-C-Methyl-D-erythritol-4-phosphate (MEP) pathway in plastids) (Ma et al., 2012). The universal diterpenoid precursor, geranylgeranyl diphosphate (GGPP), is then biosynthesized GGPP synthase (GGPPS) (Shi et al., 2016a,b). Then, three known synthases, namely, copalyl diphosphate synthase 1 (CPS1), kaurene synthase-like 1 (KSL1), and miltiradiene oxidase (CYP76AH1), and a still-unidentified enzyme(s) produce tanshinones (Gao et al., 2009; Xing et al., 2018). Overexpression or downregulation of one or two synthases significantly impacts tanshinone synthesis (Kai et al., 2011; Wang et al., 2015; Ma et al., 2016). Plant transcription factors (TFs) play important regulatory roles in resistance to stress and metabolic engineering. TFs can regulate one or multiple biosynthetic genes from a single or multiple pathways (Xu et al., 2016; Wang et al., 2017; Yang et al., 2017). TFs are a diverse group of genes including many families and subfamilies, such as bHLH, MYB, ERF, bZIP, and GRAS (Deng et al., 2020a,b; Hao et al., 2020; Shi et al., 2021a,b; Zhou et al., 2021a,b). Less is known about the regulatory actions or target genes of the GRAS TFs, which play a critical role in the tanshinone biosynthetic pathway in *S. miltiorrhiza*.

The GRAS TF family has been discovered to be unique in plants. GRAS TFs consist of three gene members, namely, gibberellic acid insensitive (GAI), repressor of GA1-3 mutant (RGA), and Scarecrow (*SCR*). A whole-genome analysis from *Arabidopsis thaliana*, rice (*Oryza sativa*), and musk lily (*Lilium longiflorum*) grouped the GRAS families into ten distinctive subfamilies, namely, *SCR*, short-root (*SHR*), *L. longiflorum* SCR-like (*LISCL*), *DELLA*, SCL-like 3 (*SCL3*), SCL-like 4 and SCL-like 7 (*SCL4/7*), *A. thaliana* Lateral suppressor (*AtLAS*), hairy meristem (*HAM*), *A. thaliana* phytochrome A signal transduction 1 (*AtPAT1*), and dwarf and low tillering (*DLT*) (Sun et al., 2011). The SCR and SHR subfamilies were validated to act as positive regulators to promote the formation of root radial morphology and root monolayer cells in *Arabidopsis* (Koizumi et al., 2012; Rich et al., 2017). SCR is mainly expressed in root epidermal cells regulating the division of daughter cells differentiation in root, whereas SHR not only affects the polar differentiation of root endothelial cells but also activates the SCR promoter in specific tissues to regulate its function (Slewinski et al., 2012). In addition, SHR proteins are expressed explicitly in the mid-column sheath cells. They can transfer from external monolayers to endothelial cells to form a dimer with SCR, which jointly activates the transcription of the *SCR* gene and other downstream target genes. Therefore, it can be inferred that SCR and SHR are transcriptionally dependent on each other, and the resulting SCR-SHR complex positively modulates the transcription of SCR (Koizumi et al., 2012; Slewinski et al., 2012). Yeast extract (YE), also known as yeast flavoring, plays a vital role in promoting the accumulation of secondary metabolites in plants. In *S. miltiorrhiza*, after treating hairy root culture with YE, the content of tanshinone was significantly increased (Chen et al., 2001). However, the transcriptional mechanism involved in YE-triggered tanshinone biosynthesis remains obscure.

In this study, we isolated and identified a new GRAS TF named *SmSCR1*. Our studies uncover the regulatory role of *SmSCR1* gene in response to YE-induced regulation of tanshinone biosynthesis and hairy root growth and development. Our data discover a novel TF family, GRAS, as a candidate for improving the biosynthesis potential of tanshinone through metabolic engineering strategies.

## Materials and Methods

### Plant Materials and Growth Conditions

*Salvia miltiorrhiza* plants were grown in solid Murashige and Skoog (MS) medium at 25°C, in 60% humidity, and in a light rhythm of 16-h light and 8-h dark (Shi et al., 2021a,b). *S. miltiorrhiza* hairy roots were subcultured in 250 ml Erlenmeyer flasks with 100 ml 1/2 MS liquid medium and placed in a shaker at 110 rpm min^–1^, 25°C in the dark. The hairy roots were collected after 60 days for gene expression analysis (Shi et al., 2016a,^2020^). *Nicotiana benthamiana* was directly sown in soil and cultivated in pots for further experimental needs (Huang et al., 2019). RNA was extracted from different tissues of annual plants of *S. miltiorrhiza* and stored at −80°C using liquid nitrogen flash freezing.

### Gene Isolation and Sequence Analysis

All the GRAS families in *S. miltiorrihiza* were identified against local datasets. *SmSCR1*, which belongs to the GRAS family and intensively responds to YE induction, was cloned using a homology-based cloning method, as described previously (Zhou et al., 2016, 2017). The cloned *SmSCR1* gene was analyzed using the BlastP tool in the NCBI database. The reported GRAS members, i.e., SmGRAS1 (accession number: KY435886) and SmGRAS2 (KY435887) in *S. miltiorrihiza*, together with *O. sativa* OsGRAS32 (Os06g0127800), *O. sativa* OsSCR1 (XP_015615402.1), *O. sativa* OsSCR2 (XP_015620600.1), *A. thaliana* AtSCR (Q9M384.1), and *A. thaliana* AtSCL23 (NM_123557), which are highly homologous to *SmSCR1* (OM032820), were subjected to amino acid sequence alignment using Vector NTI software (Invitrogen, Carlsbad, CA, United States). The full-length amino acid sequences of the GRAS were aligned using the neighbor-joining (NJ) method in the Clustal X computer, and the MEGA 6.0 program was used to generate a phylogenetic tree. Using Clustal X computing tools, the amino acid sequences of candidate GRAS family members were aligned based on the NJ method, and MEGA 6.0 software was used to generate phylogenetic trees. Subfamily members of SCR and SCL from *Arabidopsis* and rice were compared with *SmSCR1* using Vector NTI 10.0 software.

### Subcellular Localization Analysis of *SmSCR1*

To verify the subcellular localization profile of *SmSCR1*, pHB*-SmSCR1-YFP*, and pHB*-YFP* recombinant plasmids were introduced into *Agrobacterium rhizogenes* strain EHA105 for transient transformation, respectively. pHB-YFP was used as the positive control. After 48 h of infection by *Agrobacterium tumefaciens*, YFP signals from infected *N. benthamiana* leaves were visualized using a high-resolution microscope observation system. The nuclei of epidermal cells of infected *N. benthamiana* leaves were stained with 4′, 6-diamidino-2-phenylindole dihydrochloride (DAPI) solution for 3 h before observation (Zhou et al., 2020).

### Quantitative Real-Time PCR

According to the procedure reported before (Shi et al., 2016b), the quantitative real-time PCR (qRT-PCR) analysis was conducted. Different tissues (e.g., taproot, lateral root, fibrous root, phloem, xylem, stem, petiole, young leaves, and mature leaves) were taken from an adult plant, as well as hairy roots with the treatment of YE, and then frozen in liquid nitrogen for RNA isolation (Zhang et al., 2011). *Actin* gene of *S. miltiorrhiza* was used as the internal reference. The sequences of all primer pairs used in qRT-PCR studies are summarized in Supplementary Table 1. The gene expression was quantified by the comparative *Ct* method (Hao et al., 2015).

### Transformation of *SmSCR1* in *Salvia miltiorrhiza*

The open reading frame (ORF) of *SmSCR1* was introduced into the double restriction insertion site of *Bam*HI and *Spe*I of the pHB vector under the CaMV 35S promoter and NOS terminator (Supplementary Figure 2A). The DNA sequence encoding the SRDX structural domain (LDLDLELRLGFA) was inserted into the ORF of *SmSCR1* just before the stop codon (TAA) using the method described previously (Hiratsu et al., 2003; Deng et al., 2020a,b). The SmSCR1-SRDX was then subcloned into the pHB to create the pHB-SmSCR1-SRDX suppressive expression construct (Supplementary Figure 2B). To obtain the transgenic hairy roots, all plasmids were transformed into *A. tumefaciens* C58C1 and then transinfected into *S. miltiorrhiza* (Zhou et al., 2016).

### Determination of Tanshinones by High-Performance Liquid Chromatography

After 60 days of continuous culture in 1/2 MS liquid medium, each hairy root line was harvested and dried in a freeze dryer for 24 h, and then extracted with 16 ml methanol/dichloromethane (3:1, v/v). Notably, 100 mg of dried powder of *S. miltiorrhiza*, sonicated for 1 h, then soaked overnight in the dark and centrifuged for 10 min at 12,000 × *g*. After centrifugation, the supernatant was poured into a distillation flask. After centrifugation, the supernatant was poured into a distillation flask and spun dry at 50°C. We then added 2 ml of methanol to dissolve the material in the distillation flask. After further centrifugation at 6,500 × *g* for 10 min, the samples were filtered separately through 0.22 μM organic membranes (Jinten, China) using a 1 ml sterile syringe (Huang et al., 2019). All metabolites were quantified by high-performance liquid chromatography (HPLC), as described previously (Agilent Technologies, Santa Clara, CA, United States) (Zhou et al., 2016; Sun et al., 2019; Yang et al., 2021).

### Dual-Luciferase Assay

The dual-luciferase (Dual-LUC) assays were carried out as previously reported (Deng et al., 2019). The recombinants of pHB-YFP and pHB-SmSCR1-YFP were introduced into *A. tumefaciens* GV3301,respectively. Gene promoters from the tanshinone biosynthetic pathway were cloned and inserted into pGREEN0800 vector, respectively, which were co-transformed into the GV3301 strain with pSoup19. The test was carried out as previously reported (Cao et al., 2018). The pHB-YFP construct was employed as the negative control, and the *Renilla* gene was used as the internal control.

### Yeast One-Hybrid Assay

Similar to a previous study (Deng et al., 2019), the ORF of *SmSCR1* was inserted into the pB42AD vector. Three fragments of SmCPS1 promoter sequence (i.e., from −1 to −700, −701 to −1,400, and −1,401 to −2,100 bp, relative to translation start site, respectively) were inserted into a pLacZ plasmid. The resulting recombinants were co-transformed into yeast cell EGY48a. After cultivation on SD/-Ura/-Leu medium for 48 h, the positive binding activity was examined on SD/-Ura/-Leu medium with X-gal for 24 h. PB42AD and pLacZ empty carriers were set as negative controls.

### Morphological Observations of *SmSCR1*

To investigate the effect of the *SmSCR1* gene on plant growth and development, we examined different phenotypes of the transgenic hairy roots on 1/2 MS medium. In addition, three different transgenic hairy roots of *SmSCR1* with good growth and similar developmental time were selected for developmental microstructure observation, as reported previously (Wu et al., 2020). The pith (Pi) and posterior xylem (Mx) of the hairy roots were observed under a light microscope (200 ×).

### Statistical Analysis

Each data represent the average of three independent experimental calculations, and the data are expressed as the mean ± SD. SPSS 16.0 software was used to perform a single-sample *t*-test and one-way ANOVA, and *p*-values < 0.05 were considered statistically significant.

## Results

### Isolation and Sequence Analysis of *SmSCR1* Gene

**FIGURE 1:**
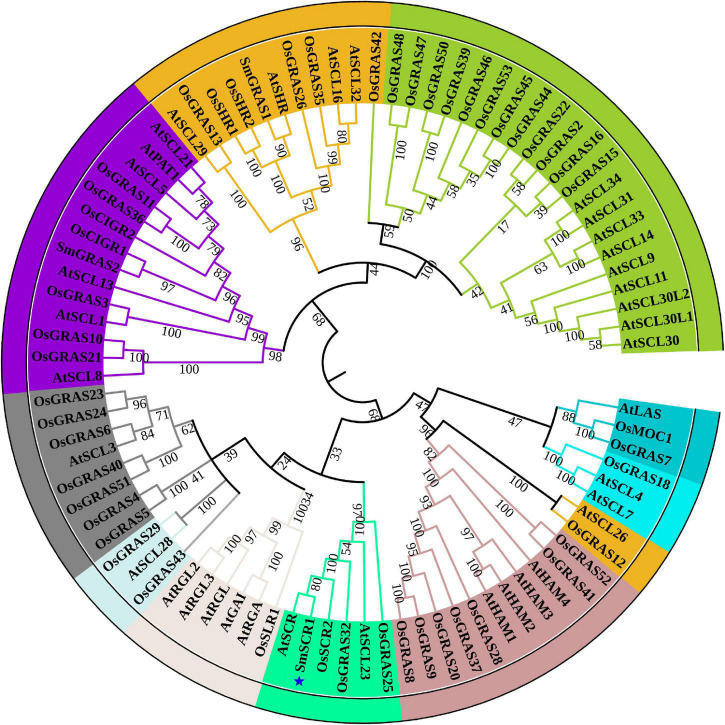
Bioinformatics analysis of *SmSCR1*. Phylogenic tree analysis of *SmSCR1* and GRAS transcription factors from different species. The MEGA 6.05 software was employed to construct the phylogenetic tree with the amino acid sequences of *SmSCR1* in *Salvia miltiorrhiza* and 89 GRAS family members in *Arabidopsis* and rice using the neighbor-joining approach. The bootstrap values after 1,000 replicates are indicated by the numbers on the nodes.

**FIGURE 2:**
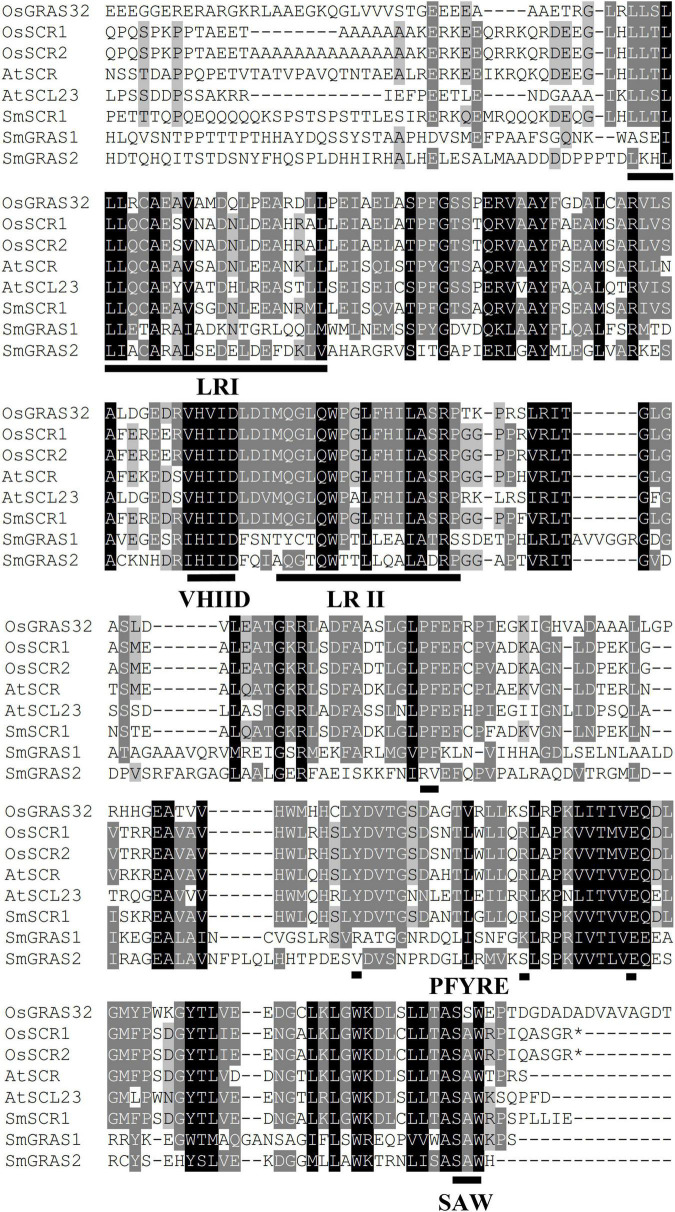
Multiple alignments of *SmSCR1* with related GRAS proteins from other plants. SmGRAS1 (accession number: KY435886), SmGRAS2 (KY435887), OsGRAS32 (Os06g0127800), OsSCR1 (XP_015615402.1), OsSCR2 (XP_015620600.1), AtSCR (Q9M384.1), AtSCL23 (NM_123557), and *SmSCR1* (OM032820). Black line indicates the conserved amino acid sites. ILR I, leucine-rich region I; LR II, leucine-rich region II; VHIID, the conserved region consisting of valine, histidine, isoleucine, and D-aspartic acid; PFYRE, the conserved amino acid sites consisting of proline, phenylalanine, tyrosine, arginine, and glutamic acid; SAW, the conserved region consisting of serine, alanine, and tryptophan.

First, we identified a *GRAS* TF (unigene number: SmiContig9465) and *SmSCR1* (NCBI accession number: OM032820), responding to YE-induction (fold change > 2) from the *S. miltiorrhiza* transcriptome (Zhou et al., 2017). *SmSCR1* length is 1,944 bp long and encodes 647 amino acids, with a molecular weight size of 70.515 kDa coupled with a theoretical isoelectric point of 5.78. GRAS family members from *Arabidopsis* and rice were analyzed with *SmSCR1* to construct a phylogenetic tree. As shown in Figure 1, *SmSCR1* has the highest relationship with *AtSCR* in *Arabidopsis* and *OsSCR2* in rice, implying that *SmSCR1* belongs to the *SCR* subgroup of the *GRAS* family. *SmSCR1* consists of several conserved motifs, including VHIID, PFYRE, SAW motifs, and the leucine-rich regions (LRs), i.e., LR I and LR II (Figure 2).

### Expression Analysis of *SmSCR1*

**FIGURE 3:**
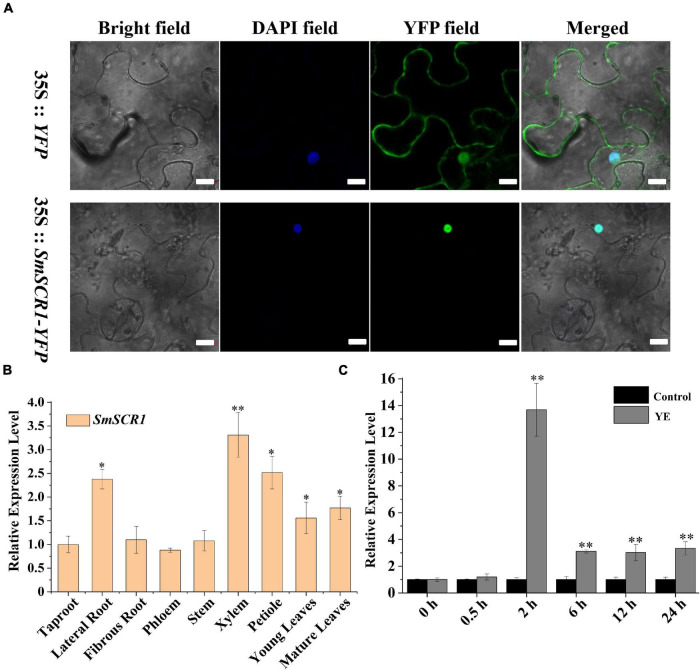
Expression profile of *SmSCR1*. **(A)** Transient expression of the *SmSCR1* in *Nicotiana benthamiana* leaf epidermal cells. Scale bars represent 10 μm. **(B)** The expression patterns of *SmSCR1* in different tissues. Fold changes of the relative expression level of *SmSCR1* gene in other tissues are all normalized to the taproot. **(C)** Induced effect of YE on the expression of *SmSCR1*. Fold changes of the gene relative expression levels are all normalized to the control without induction by YE at 0 h. Asterisks indicate significant differences between the taproot and other tissues in **(B)** and between treatment and control in **(C)** at one significant level of *t*-test (*p < 0.05; **p < 0.01). Data are means of three replicates with SDs.

Transient expression of *SmSCR1* in epidermal cells from 45-day-old *N. benthamiana* leaves exhibited that *SmSCR1* located in the nucleus (Figure 3A). The gene expression of *SmSCR1* was characterized from nine different tissues of *S. miltiorrhiza* by qRT-PCR. *SmSCR1* was expressed in all tissues, namely, taproot, lateral root, fibrous root, phloem, xylem, stem, petiole, young leaves, and mature leaves, with the highest expression in xylem and petiole, and the lowest expression in taproot (Figure 3B). The expression of *SmSCR1* after YE-induction was examined, reaching the highest levels for 2 h after post-induction with 13.6-fold compared to the control (Figure 3C).

### *SmSCR1* Activates the Transcription of *Copalyl Diphosphate Synthase 1* Gene

**FIGURE 4:**
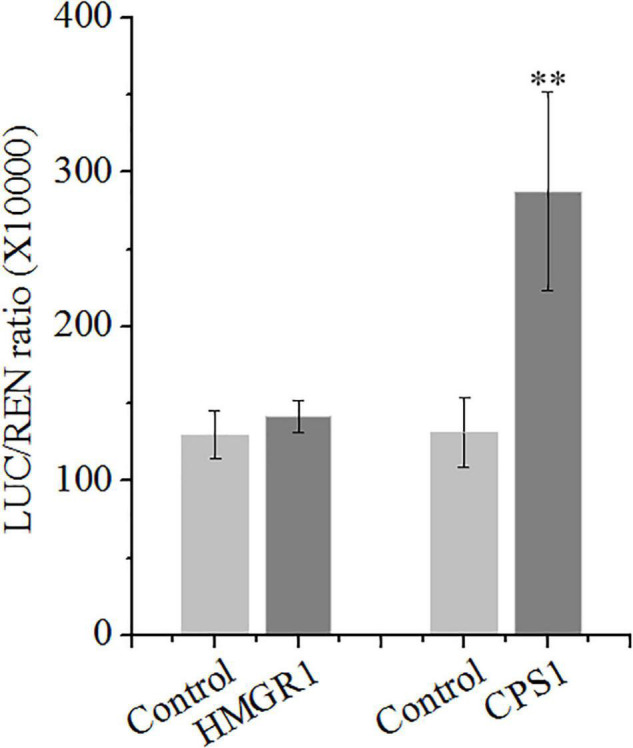
*SmSCR1* activates the transcription of *SmCPS1*. *SmSCR1* activates the promoter of *SmCPS1* by the dual-luciferase (Dual-LUC) assay. The SmCPS1 promoter was fused to the LUC reporter, and its activity was determined by a transient Dual-LUC assay in *Nicotiana benthamiana*. The value of LUC/REN was recorded as the activating activity. Asterisk indicates significant differences at one significant level of *t*-test (**p < 0.01). Data are means of three replicates with SDs.

The role of *SmSCR1* in regulating tanshinone biosynthetic gene expressions was examined. The Dual-LUC assay showed that *SmSCR1* uniquely activates the transcription of the *SmCPS1* promoter, leading to a 2.19-fold increase compared with the control, but not to other promoters (e.g., *HMGR1* promoter) (Figure 4). Y1H assay showed that self-activating of pB42AD vector binding to the promoter of the *CPS1* gene is visible (Supplementary Figure 3). Those results indicated that *SmSCR1* activated the transcription of *SmCPS1* to increase the production of tanshinone in *S. miltiorrhiza*.

### *SmSCR1* Promotes Tanshinone Biosynthesis in the Transgenic Hairy Root of *Salvia miltiorrhiza*

**FIGURE 5:**
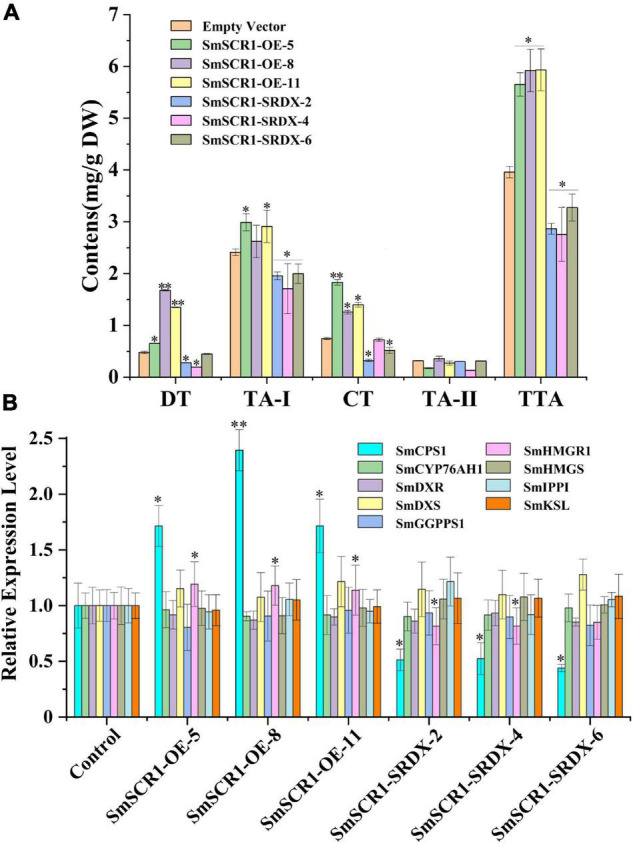
Tanshinone content and gene expression in the tanshinone biosynthetic pathway in *Salvia miltiorrhiza* transgenic hairy root lines. **(A)** High-performance liquid chromatography analysis of tanshinone content in hairy root lines of SmSCR1-OE (overexpression) and SmSCR1-SRDX (suppression). DT, dihydrotanshinone; CT, cryptotanshinone; TA-I, tanshinone I; TA-II, tanshinone IIA; TTA, total tanshinone. **(B)** Gene expression in the SmSCR1-OE and SmSCR1-SRDX lines for the underlying tanshinone biosynthesis. The blank vector without *SmSCR1* gene is used as the control to standardize fold differences in relative gene expression levels in transgenic hairy roots. The standard errors of the mean are represented by error bars. The asterisks on the bar indicate significant differences by *t*-test compared to the control at two significant levels (*p < 0.05; **p < 0.01).

The OE and SRDX suppressive hairy root lines were first examined by genomic PCR detection (Supplementary Figure 4). Then, the transcript profiles of *SmSCR1* in transgenic hairy roots were quantified by qRT-PCR. Three independent OE lines (i.e., OE-5, OE-8, and OE-11) and three SRDX lines (i.e., SRDX-2, SRDX-4, and SRDX-6) of *SmSCR1* with the highest expression levels in transgenic lines were subsequently chosen for further analysis (Supplementary Figure 5). After 60-day-old subculture, the hairy root lines were collected independently and were subjected to examine the content of tanshinones by HPLC (Figure 5A). The concentrations of DT, TA-1, CT, and TTA were significantly increased compared to the control in the OE lines, with the highest total tanshinone (TTA) content in SmSCR1-OE-11, reaching 1.49-fold than that of the control. In contrast, in the SRDX repression lines, DT, TA-1, CT, and TTA were significantly decreased compared to the control, with the lowest TTA content of 2.76 mg/g DW. The above results suggest that the *SmSCR1* TF can promote the tanshinone accumulation in *S. miltiorrhiza*.

### *SmSCR1* Upregulates *Copalyl Diphosphate Synthase 1* and *HMGR1* in Transgenic Hairy Roots of *Salvia miltiorrhiza*

Copalyl diphosphate synthase 1 and *HMGR1* were significantly upregulated in OE lines (e.g., OE-5, OE-8, and OE-11), with the SmSCR1-OE-11 line, showing the highest expression. In contrast, the two genes were drastically downregulated in the three SRDX lines (i.e., 2, 4, and 6), with the SmSCR1-SRDX-6 line exhibiting the lowest decrease compared to the control (Figure 5B). These findings showed that *SmSCR1* stimulated *CPS1* and *HMGR1* together, resulting in increased production in transgenic hairy roots of *S. miltiorrhiza*.

### *SmSCR1* Promotes the Growth of Transgenic Hairy Roots of *Salvia miltiorrhiza*

**FIGURE 6:**
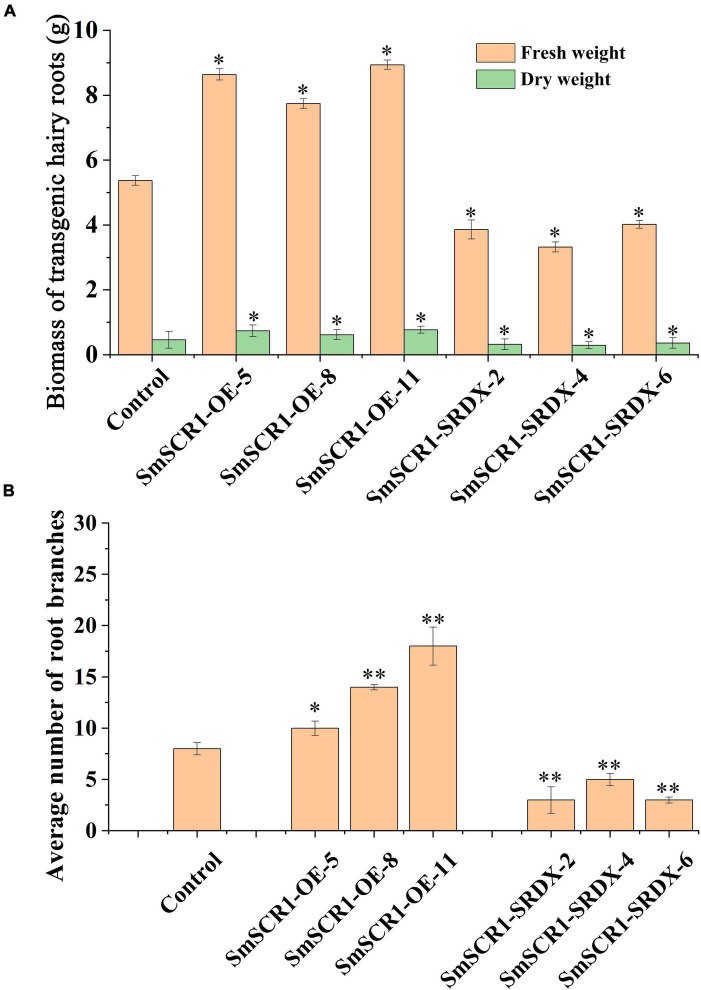
Phenotype of the *SmSCR1* transgenic hairy root. **(A)** Biomass of transgenic hairy root. **(B)** An average number of root branches. Asterisks indicate significant differences between the transgenic hairy root lines and control at one significant level of *t*-test (*p < 0.05; **p < 0.01). Data are means of three replicates with SDs.

The biomass of the transgenic hairy root lines significantly increased in the *SmSCR1* OE lines and significantly reduced in the SRDX lines compared to the control (Figure 6A). Moreover, the *SmSCR1* overexpression hairy root lines had a better growth status than the control, with more branches in the *SmSCR1* OE lines and fewer in the SRDX suppressive hair roots (Figure 6B). Roots are formed by a series of asymmetrical divisions of early differentiated cells to produce the inner and outer cortex of the root, which then develops into xylem and phloem. The development of transgenic hairy roots by paraffin sectioning demonstrated that the *SmSCR1* gene promotes the formation of hairy root radiation morphology of the Mx cell and Pi cell differentiation in *SmSCR1*-OE hairy roots when compared with the control and the SRDX suppressive hairy roots (Figure 6). Those results suggest that *SmSCR1* can modulate the growth and development of hairy roots in *S. miltiorrhiza*.

## Discussion

The GRAS TF family is an important TF class involved in regulating plant growth, development, environmental stress response, and growth signal transduction (Sun et al., 2011). With the development of genome sequencing technology, a few GRAS family members have been identified in plants, such as plum (*Armeniaca mume*), lotus (*Nelumbo nucifera*), and pine blue (*Isatis indigotica*) (Kamiya et al., 2003; Hou et al., 2010). However, only a few reports systematically characterized GRAS TFs in medicinal plants. We identified a novel YE-responsive GRAS TF gene, *SmSCR1* gene, encoding 647 amino acids with several key GRAS conserved structural domains (e.g., VHIID, LR I, LR II, PFYRE, and SAW motifs) (Koizumi et al., 2011; Zhou et al., 2017). In a previous study, SCR and SHR families were thought to co-regulate the plant growth, development, and radial structures in roots (Koizumi et al., 2011, 2012; Fan et al., 2017). We also identified that *SmSCR1* was very sensitive to YE-induction (Figure 3C), regulating tanshinone accumulation in *S. miltiorrhiza* (Zhou et al., 2017, 2021a).

We revealed that *SmSCR1* upregulates tanshinone biosynthesis in *SmSCR1* suppressive hairy root lines (Figure 5A). Recently, two GRAS families, including *SmGRAS1* (GenBank accession number: KY435886) and *SmGRAS2* (GenBank accession number: KY435887), clustered into SHR and PAT1 subgroups, respectively (Figure 1), were characterized in *S. miltiorrhiza*. SmGRAS1 and SmGRAS2 were verified by transgenic validation to upregulate tanshinone accumulation in hairy roots. Therefore, many GRAS families might play a more prominent role in regulating tanshinone accumulation in *S. miltiorrhiza* and require further investigation.

Both *CPS1* and *HMGR1* positively correlated to the expression of *SmSCR1* in the transgenic lines, while *SmSCR1* can only bind to the *CPS1* promoter to activate its expression as examined. Due to the limited data about the specific *cis*-element in promoter binding by SCR1 in other plants (Li et al., 2019), further examining the direct binding of *SmSCR1* to the specific promoter elements by EMSA assay *in vitro* would be unfeasible. In this study, overexpressing *SmSCR1* upregulated not only the expression of its target gene *CPS1* but also the *HMGR1* gene in the tanshinone biosynthetic pathway. Overexpressing *SmERF1L1* promotes the expression of five genes in the tanshinone biosynthetic pathway, but only *DXR* is a target (Huang et al., 2019). In SmERF128 transgenic hairy roots, the expression of six genes in the tanshinone biosynthetic pathway was activated, and only three of the six genes were validated to be targets of SmERF128 (Zhang et al., 2019). Thus, it can be inferred that the ectopic expression of certain TF genes upregulates the expression of not only their target genes but also other genes in the same biosynthetic pathway.

The root formation is a series of asymmetric divisions of stem cells to generate the inner and outer cortex of the root and then differentiate into different forms of tissue. In *A. thaliana*, SCR is mainly expressed in root epidermal cells. As a positive regulator, SCR regulates root radiation morphology and plays a vital role in root monolayer cell formation (Koizumi et al., 2012; Rich et al., 2017). When SCR function is lost and mutated, the normal development of the cell layer will be affected. In addition, SCR can also regulate the division of progenitor cells and promote the growth of biomass (Koizumi et al., 2011; Slewinski et al., 2012). In rice, *OsSCR* was thought to affect the development of plant leaf guard cells (Hou et al., 2010). In the three *SmSCR1* OE lines, we found that *SmSCR1* significantly promoted the biomass increase of the three transgenic hairy root lines, whereas a visible decrease was observed in *SmSCR1* SRDX lines.

**FIGURE 7:**
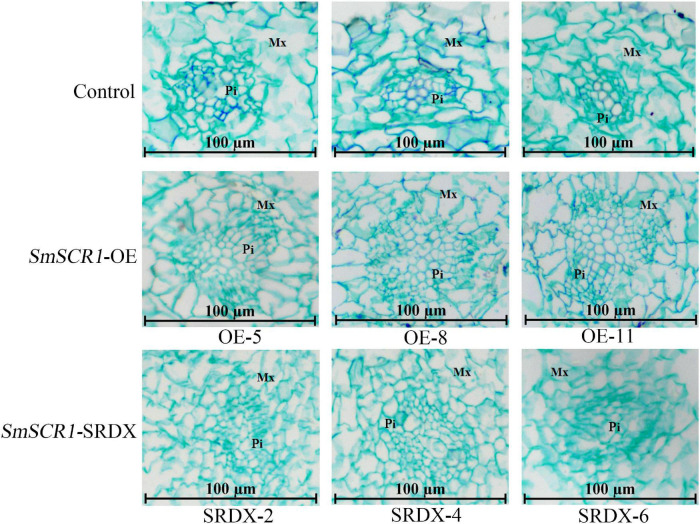
Paraffin sectioning analysis of the transgenic hairy roots. The control means the transgenic hairy roots with the transformation of pHB-X-YFP blank vector. pHB-SmSCR1 represents the overexpression lines, and pHB-SmSCR1-SRDX represents the suppression lines. Pi represents the pith. Mx represents the posterior xylem.

Moreover, *SmSCR1* can regulate the development of Pi and the Mx. In *SmSCR1* OE lines, the cell division rate of progeny and the root radial structure growth rate were significantly higher than that of control. In contrast, the three SRDX suppressive hairy root lines have an opposite phenotype (Figure 7). The function of *SmSCR1* to regulate the growth and development of hairy roots in *S. miltiorrhiza* is similar to the model plants, e.g., *Arabidopsis* and rice.

**FIGURE 8:**
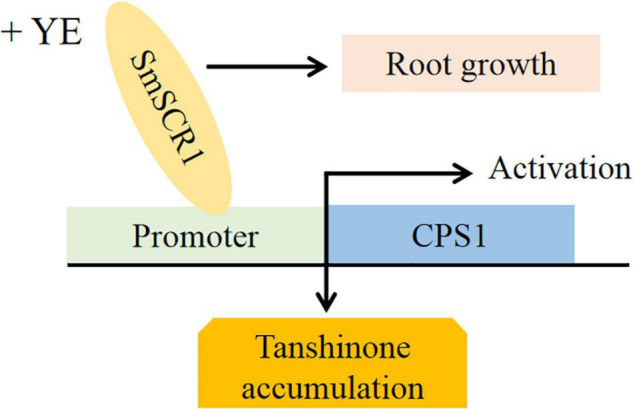
A proposed model for the role of *SmSCR1* in modulating tanshinone biosynthesis and root growth in *Salvia miltiorrhiza*.

Based on the present findings, a deductive model of *SmSCR1* as an activator in response to YE-induction promotes tanshinone accumulation, Pi cell division, radial structure formation, and biomass increase of hairy roots in *S. miltiorrhiza* was outlined (Figure 8).

## Data Availability Statement

The original contributions presented in the study are included in the article/Supplementary Material, further inquiries can be directed to the corresponding author.

## Author Contributions

GK and WZ conceived and designed the project. SW, YS, YL, XG, CC, and TL prepared materials and performed the experiments. WZ, SW, YS, IM, YL, and CW performed the bioinformatics analysis and analyzed the data. GK, WZ, SW, and YS wrote the manuscript. IM, WZ, and GK revised the manuscript. All authors read and approved the final version of the manuscript.

## Conflict of Interest

The authors declare that the research was conducted in the absence of any commercial or financial relationships that could be construed as a potential conflict of interest.

## Publisher’s Note

All claims expressed in this article are solely those of the authors and do not necessarily represent those of their affiliated organizations, or those of the publisher, the editors and the reviewers. Any product that may be evaluated in this article, or claim that may be made by its manufacturer, is not guaranteed or endorsed by the publisher.

## Supplementary Material

Supplementary Figure 1 The skeleton of tanshinone biosynthetic pathway in *Salvia miltiorrhiza*.

Supplementary Figure 2 Recombinants of *SmSCR1*-overexpressive and suppressed vectors. **(A)** Recombinant vector of pHB *-SmSCR1*-YFP. **(B)** Recombinant vector of pHB-*SmSCR1*-SRDX.

Supplementary Figure 3 Yeast one-hybrid assay of *SmSCR1* binding to the SmCPS1 promoter. P1, P2, and P3 represent the three fragments of *SmCPS1* promoter sequence from −1 to −700, −701 to −1,400, and −1,401 to −2,100 bp, relative to translation start site, respectively.

Supplementary Figure 4 Detection of the expression level of *SmSCR1* gene in transgenic hairy root lines by qRT-PCR.

Supplementary Figure 5 Identification of transgenic hairy root lines by PCR. The asterisks on the bar indicate significant differences by *t*-test compared to the control (***P* < 0.01).

## Abbreviations

CPS1: copalyl diphosphate synthase I
HMGR1: 3-hydroxy-3-methylglutaryl-CoA-reductase I
GGPPS1: geranylgeranyl diphosphate synthase I
HMGS: hydroxymethylglutaryl-CoA synthase
IPPI: isopentenyl diphosphate isomerase
KSL: ent-kaurene synthase like
CPS1: copalyl diphosphate synthase I
CYP76AH1: miltiradiene oxidase
DXR: 1-deoxy-D-xylulose 5-phosphate reductoisomerase
DXS: deoxy-D-xylulose 5-phosphate synthase II
Dual-LUC: dual-luciferase
YE: yeast extract
GRAS1: GRAS transcriptional factor 1
DT: dihydrotanshinone
CT: cryptotanshinone
TA-I: tanshinone I
TA-II: tanshinone IIA
TTA: total tanshinone.

## References

[B1] Cao W. Wang Y. Shi M. Hao X. Zhao W. Wang Y. (2018). Transcription factor SmWRKY1 positively promotes the biosynthesis of tanshinones in *Salvia miltiorrhiza*. *Front. Plant Sci.* 9:554. 10.3389/fpls.2018.00554 29755494 PMC5934499

[B2] Chen H. Chena F. Chiu F. C. Lo C. M. (2001). The effect of yeast elicitor on the growth and secondary metabolism of hairy root cultures of *Salvia miltiorrhiza*. *Enzyme Microb. Tech.* 28 100–105. 10.1016/s0141-0229(00)00284-2 11118603

[B3] Deng C. Hao X. Shi M. Fu R. Wang Y. Zhang Y. (2019). Tanshinone production could be increased by the expression of SmWRKY2 in *Salvia miltiorrhiza* hairy roots. *Plant Sci.* 284 1–8. 10.1016/j.plantsci.2019.03.007 31084862

[B4] Deng C. Shi M. Fu R. Zhang Y. Wang Q. Zhou Y. (2020a). ABA-responsive transcription factor bZIP1 is involved in modulating biosynthesis of phenolic acids and tanshinones in *Salvia miltiorrhiza*. *J. Exp. Bot.* 71 5948–5962. 10.1093/jxb/eraa295 32589719

[B5] Deng C. Wang Y. Huang F. Lu S. Zhao L. Ma X. (2020b). SmMYB2 promotes salvianolic acid biosynthesis in the medicinal herb *Salvia miltiorrhiza*. *J. Integr. Plant Biol.* 62 1688–1702. 10.1111/jipb.12943 32343491

[B6] Fan S. Zhang D. Gao C. Zhao M. Wu H. Li Y. (2017). Identification, classification, and expression analysis of GRAS gene family in *Malus domestica*. *Front. Physiol.* 8:253. 10.3389/fphys.2017.00253 28503152 PMC5408086

[B7] Fu L. Han B. Zhou Y. Ren J. Cao W. Patel G. (2020). The anticancer properties of tanshinones and the pharmacological effects of their active ingredients. *Front. Pharmacol.* 11:193. 10.3389/fphar.2020.00193 32265690 PMC7098175

[B8] Gao W. Hillwig M. L. Huang L. Cui G. Wang X. Kong J. (2009). A functional genomics approach to tanshinone biosynthesis provides stereochemical insights. *Org. Lett.* 11 5170–5173. 10.1021/ol902051v 19905026 PMC2776380

[B9] Hao X. Pu Z. Cao G. You D. Zhou Y. Deng C. (2020). Tanshinone and salvianolic acid biosynthesis are regulated by SmMYB98 in *Salvia miltiorrhiza* hairy roots. *J. Adv. Res.* 23 1–12. 10.1016/j.jare.2020.01.012 32071787 PMC7016019

[B10] Hao X. Shi M. Cui L. Xu C. Zhang Y. Kai G. (2015). Effects of methyl jasmonate and salicylic acid on tanshinone production and biosynthetic gene expression in transgenic *Salvia miltiorrhiza* hairy roots. *Biotechnol. Appl. Bioc.* 62 24–31. 10.1002/bab.1236 24779358

[B11] Hiratsu K. Matsui K. Koyama T. Ohme-Takagi M. (2003). Dominant repression of target genes by chimeric repressors that include the EAR motif, a repression domain, in *Arabidopsis*. *Plant J.* 34 733–739. 10.1046/j.1365-313x.2003.01759.x 12787253

[B12] Hou X. Lee L. Xia K. Yan Y. Yu H. (2010). DELLAs modulate jasmonate signaling via competitive binding to JAZs. *Dec. Cell* 19 884–894. 10.1016/j.devcel.2010.10.024 21145503

[B13] Huang Q. Sun M. Yuan T. Wang Y. Shi M. Lu S. (2019). The AP2/ERF transcription factor SmERF1L1 regulates the biosynthesis of tanshinones and phenolic acids in *Salvia miltiorrhiza*. *Food Chem.* 274 368–375. 10.1016/j.foodchem.2018.08.119 30372953

[B14] Jia Q. Zhu R. Tian Y. Chen B. Li R. Li L. (2019). *Salvia miltiorrhiza* in diabetes: a review of its pharmacology, phytochemistry, and safety. *Phytomedicine* 58:152871. 10.1016/j.phymed.2019.152871 30851580

[B15] Jung I. Kim H. Moon S. Lee H. Kim B. (2020). Overview of *Salvia miltiorrhiza* as a potential therapeutic agent for various diseases: an update on efficacy and mechanisms of action. *Antioxidants* 9:857. 10.3390/antiox9090857 32933217 PMC7555792

[B16] Kai G. Xu H. Zhou C. Liao P. Xiao J. Luo X. (2011). Metabolic engineering tanshinone biosynthetic pathway in *Salvia miltiorrhiza* hairy root cultures. *Metab. Eng.* 13 319–327. 10.1016/j.ymben.2011.02.003 21335099

[B17] Kamiya N. Itoh J. Morikami A. Nagato Y. Matsuoka M. (2003). The SCARECROW gene’s role in asymmetric cell divisions in rice plants. *Plant J.* 36 45–54. 10.1046/j.1365-313x.2003.01856.x 12974810

[B18] Koizumi K. Hayashi T. Wu S. Gallagher K. L. (2012). The SHORT-ROOT protein acts as a mobile, dose-dependent signal in patterning the ground tissue. *P. Natl. Acad. Sci. U S A.* 109 13010–13015. 10.1073/pnas.1205579109 22826238 PMC3420204

[B19] Koizumi K. Wu S. MacRae-Crerar A. Gallagher K. L. (2011). An essential protein that interacts with endosomes and promotes movement of the SHORT-ROOT transcription factor. *Curr. Biol.* 21 1559–1564. 10.1016/j.cub.2011.08.013 21924907

[B20] Li W. Bai Z. Pei T. Yang D. Mao R. Zhang B. (2019). SmGRAS1 and SmGRAS2 regulate the biosynthesis of tanshinones and phenolic acids in *Salvia miltiorrhiza*. *Front. Plant Sci.* 10:1367. 10.3389/fpls.2019.01367 31737003 PMC6831727

[B21] Ma Y. Ma X. H. Meng F. Y. Zhan Z. L. Guo J. Huang L. Q. (2016). RNA interference targeting CYP76AH1 in hairy roots of *Salvia miltiorrhiza* reveals its key role in the biosynthetic pathway of tanshinones. *Biochem. Bioph. Res. Co* 477 155–160. 10.1016/j.bbrc.2016.06.036 27291148

[B22] Ma Y. Yuan L. Wu B. Li X. Chen S. Lu S. (2012). Genome-wide identification and characterization of novel genes involved in terpenoid biosynthesis in *Salvia miltiorrhiza*. *J. Exp. Bot.* 63 2809–2823. 10.1093/jxb/err466 22291132 PMC3346237

[B23] Rich M. K. Courty P. E. Roux C. Reinhardt D. (2017). Role of the GRAS transcription factor ATA/RAM1 in the transcriptional reprogramming of arbuscular mycorrhiza in *Petunia hybrida*. *BMC Genomics* 18:589. 10.1186/s12864-017-3988-8 28789611 PMC5549340

[B24] Shi M. Gong H. Cui L. Wang Q. Wang C. Wang Y. (2020). Targeted metabolic engineering of committed steps improves anti-cancer drug camptothecin production in *Ophiorrhiza pumila* hairy roots. *Ind. Crop. Prod.* 148:112277. 10.1016/j.indcrop.2020.112277

[B25] Shi M. Du Z. Hua Q. Kai G. (2021a). CRISPR/Cas9-mediated targeted mutagenesis of bZIP2 in Salvia miltiorrhiza leads to promoted phenolic acid biosynthesis. *Ind. Crop. Prod.* 167:113560. 10.1016/j.indcrop.2021.113560

[B26] Shi M. Liao P. Nile S. H. Georgiev M. I. Kai G. (2021b). Biotechnological exploration of transformed root culture for value-added products. *Trends Biotechnol.* 39 137–149. 10.1016/j.tibtech.2020.06.012 32690221

[B27] Shi M. Luo X. Ju G. Li L. Huang S. Zhang T. (2016a). Enhanced diterpene tanshinone accumulation and bioactivity of transgenic *Salvia miltiorrhiza* hairy roots by pathway engineering. *J. Agr. Food Chem.* 64 2523–2530. 10.1021/acs.jafc.5b04697 26753746

[B28] Shi M. Zhou W. Zhang J. Huang S. Wang H. Kai G. (2016b). Methyl jasmonate induction of tanshinone biosynthesis in *Salvia miltiorrhiza* hairy roots is mediated by JASMONATE ZIM-DOMAIN repressor proteins. *Sci. Rep.* 6:20919. 10.1038/srep20919 26875847 PMC4753458

[B29] Slewinski T. L. Anderson A. A. Zhang C. Turgeon R. (2012). Scarecrow plays a role in establishing Kranz anatomy in maize leaves. *Plant Cell Physiol.* 53 2030–2037. 10.1093/pcp/pcs147 23128603

[B30] Sun M. Shi M. Wang Y. Huang Q. Yuan T. Wang Q. (2019). The biosynthesis of phenolic acids is positively regulated by the JA-responsive transcription factor ERF115 in *Salvia miltiorrhiza*. *J. Exp. Bot.* 70 243–254. 10.1093/jxb/ery349 30299490

[B31] Sun X. Xue B. Jones W. T. Rikkerink E. Dunker A. K. Uversky V. N. (2011). A functionally required unfoldome from the plant kingdom: intrinsically disordered N-terminal domains of GRAS proteins are involved in molecular recognition during plant development. *Plant Mol. Biol.* 77 205–223. 10.1007/s11103-011-9803-z 21732203

[B32] Wang B. Sun W. Li Q. Li Y. Luo H. Song J. (2015). Genome-wide identification of phenolic acid biosynthetic genes in *Salvia miltiorrhiza*. *Planta* 241 711–725. 10.1007/s00425-014-2212-1 25471478

[B33] Wang N. Xu H. Jiang S. Zhang Z. Lu N. Qiu H. (2017). MYB12 and MYB22 play essential roles in proanthocyanidin and flavonol synthesis in red-fleshed apple (*Malus sieversii f. niedzwetzkyana*). *Plant J.* 90 276–292. 10.1111/tpj.13487 28107780

[B34] Wu X. Riaz M. Yan L. Zhang Z. Jiang C. (2020). How the cells were injured and the secondary metabolites in the shikimate pathway were changed by boron deficiency in trifoliate orange root. *Plant Physiol. Biochem.* 151 630–639. 10.1016/j.plaphy.2020.04.009 32335386

[B35] Xing B. Liang L. Liu L. Hou Z. Yang D. Yan K. (2018). Overexpression of SmbHLH148 induced biosynthesis of tanshinones as well as phenolic acids in *Salvia miltiorrhiza* hairy roots. *Plant Cell Rep.* 37 1681–1692. 10.1007/s00299-018-2339-9 30229287

[B36] Xu H. Song J. Luo H. Zhang Y. Li Q. Zhu Y. (2016). Analysis of the genome sequence of the medicinal plant *Salvia miltiorrhiza*. *Mol. Plant.* 9 949–952. 10.1016/j.molp.2016.03.010 27018390 PMC5517341

[B37] Yang M. Wang Q. Liu Y. Hao X. Wang C. Liang Y. (2021). Divergent camptothecin biosynthetic pathway in *Ophiorrhiza pumila*. *BMC Biol.* 19:122. 10.1186/s12915-021-01051-y 34134716 PMC8207662

[B38] Yang N. Zhou W. Su J. Wang X. Li L. Wang L. (2017). Overexpression of SmMYC2 increases the production of phenolic acids in *Salvia miltiorrhiza*. *Front. Plant Sci.* 8:1804. 10.3389/fpls.2017.01804 29230228 PMC5708653

[B39] Zhang L. Yan X. M. Wang J. Li S. S. Liao P. Kai G. Y. (2011). Molecular cloning and expression analysis of a new putative gene encoding 3-hydroxy-3-methylglutaryl-CoA synthase from *Salvia miltiorrhiza*. *Acta Physiol. Plant.* 33 953–961. 10.1007/s11738-010-0627-2

[B40] Zhang Y. Ji A. Xu Z. Luo H. Song J. (2019). The AP2/ERF transcription factor SmERF128 positively regulates diterpenoid biosynthesis in *Salvia miltiorrhiza*. *Plant Mol. Biol.* 100 83–93. 10.1007/s11103-019-00845-7 30847712

[B41] Zhou W. Huang F. F. Li S. Wang Y. Zhou C. C. Shi M. (2016). Molecular cloning and characterization of two 1-deoxy-D-xylulose-5-phosphate synthase genes involved in tanshinone biosynthesis in *Salvia miltiorrhiza*. *Mol. Breed.* 36:124. 10.1007/s11032-016-0550-3

[B42] Zhou W. Huang Q. Wu X. Zhou Z. Ding M. Shi M. (2017). Comprehensive transcriptome profiling of Salvia miltiorrhiza for discovery of genes associated with the biosynthesis of tanshinones and phenolic acids. *Sci. Rep.* 7:10554. 10.1038/s41598-017-10215-2 28874707 PMC5585387

[B43] Zhou W. Li S. Maoz I. Wang Q. Xu M. Feng Y. (2021a). SmJRB1 positively regulates the accumulation of phenolic acid in *Salvia miltiorrhiza*. *Ind. Crop. Prod.* 164:113417. 10.1016/j.indcrop.2021.113417

[B44] Zhou W. Shi M. Deng C. Lu S. Huang F. Wang Y. (2021b). The methyl jasmonate-responsive transcription factor SmMYB1 promotes phenolic acid biosynthesis in *Salvia miltiorrhiza*. *Hortic. Res.* 8:10. 10.1038/s41438-020-00443-5 33384411 PMC7775463

[B45] Zhou Y. Sun W. Chen J. Tan H. Xiao Y. Li Q. (2020). Author correction: SmMYC2a and SmMYC2b played similar but irreplaceable roles in regulating the biosynthesis of tanshinones and phenolic acids in *Salvia miltiorrhiza*. *Sci. Rep.* 10:7201. 10.1038/s41598-020-62994-w 32332760 PMC7181698

