# Supplementary material for: Overexpression of SmSCR1 Promotes Tanshinone Accumulation and Hairy Root Growth in Salvia miltiorrhiza
Source: Front Plant Sci. 2022 Mar 8;13:860033. doi: 10.3389/fpls.2022.860033 (PMC8957878; doi:10.3389/fpls.2022.860033)
Supplement: Supplementary file 1 [file Table_1.DOCX]

**Table S1. Primer sequences used in this study**

| **Primers** | **Sequences (5’-3’)** |
| --- | --- |
| SmKSL1- 1480F | GTGTGACCCTTCTGCTAGCA |
| SmKSL1 -1630R | TGCATTGTCTTGGGAAGATG |
| SmGGPPS1 -603F | GCTGTGCTCGCAGGGGATG |
| SmGGPPS1 -774R | ATCGCCGGTGCAGTTCAGG |
| SmDXS2 -1828F | TTGGAGATTGGGAAGGGAAGGAT |
| SmDXS2 -1980R | AGGCTTGCAGAATCTCGCATCAG |
| SmHMGR-982F | TCGTTTTCAATAAGTCGAGTAGA |
| SmHMGR-1142R | ATTCTGAAGGAAGTCCAAAACAT |
| SmHMGS-282F | GATGAGCATGACAGCGGTTACTTC |
| SmHMGS-362R | GGATTTGCTCTTGTCGAGTACGGT |
| SmDXR-1248F | CGACGAGAAAATCGGATACCTGG |
| SmDXR-1424R | CATACAAGAGCAGGACTCAAACCG |
| SmIPPI-1422F | GCAACGATCCACAACTAAGGT |
| SmIPPI-1572R | ATGCCGAGTTCATCCAACAG |
| SmCPS1-F214 | ACTACCGTTCATCAAGGCCA |
| SmCPS1-R421 | CCTCGAGTTGATTCTGCACG |
| SmCYP76AH1-F221 | TCCATCTCGGCAGCCTCTACAC |
| SmCYP76AH1-R304 | GAGAAGACCTGCCCGTGCCT |
| SmActin F | AGCACCGAGCAGCATGAAGATT |
| SmActin R | AGCAAAGCAGCGAACGAAGAGT |
| NOS-R | CCCGATCTAGTAACATAGATGACA |
| RolB-F | GCTCTTGCAGTGCTAGATTT |
| RolB-R | CCCCGAGAGTCGCAGGGTTAG |
