# Supplementary figures and images for: Overexpression of SmSCR1 Promotes Tanshinone Accumulation and Hairy Root Growth in Salvia miltiorrhiza
Source: Front Plant Sci. 2022 Mar 8;13:860033. doi: 10.3389/fpls.2022.860033 (PMC8957878; doi:10.3389/fpls.2022.860033)

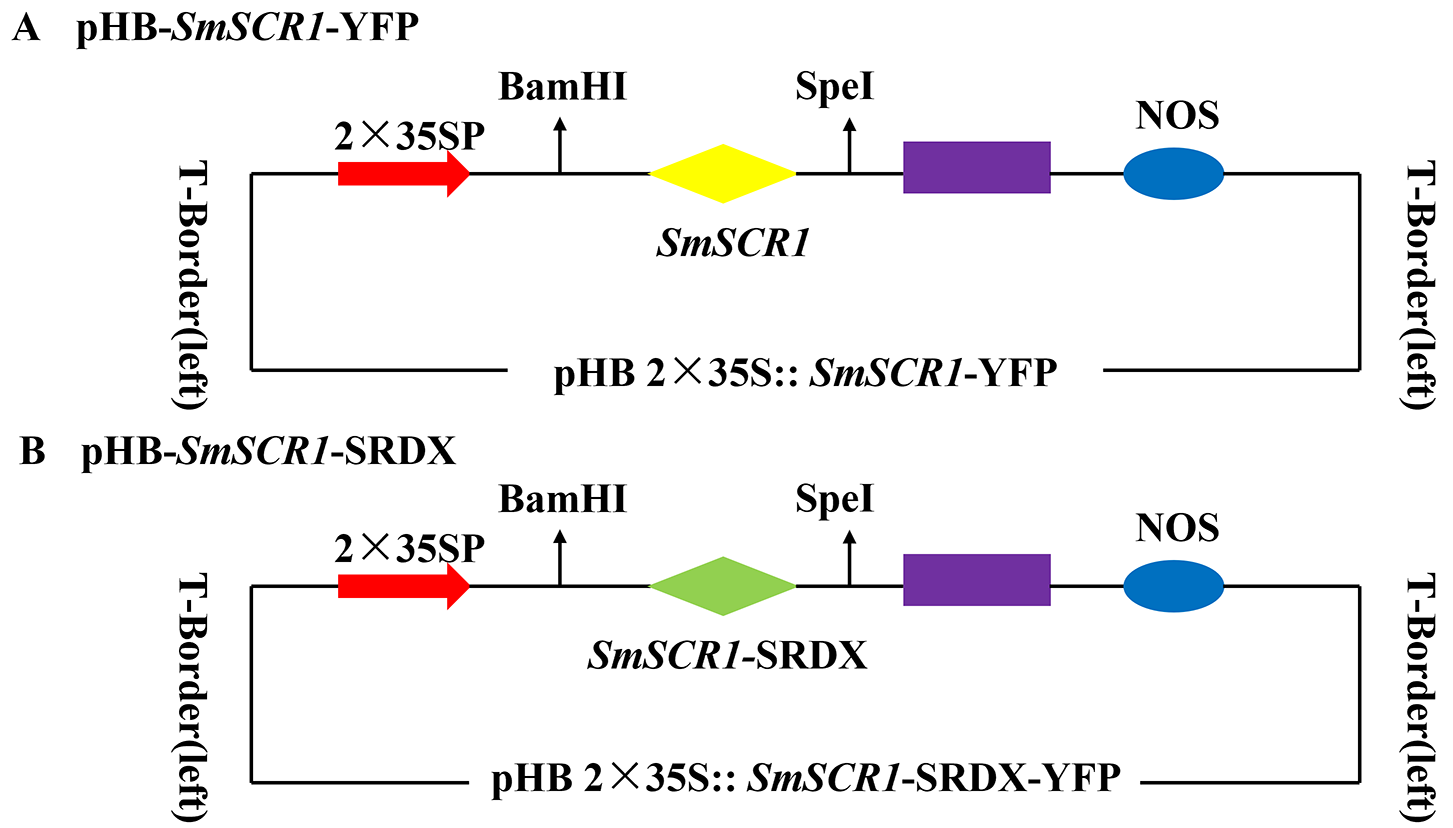

Supplement: Supplementary Figure 2 — Recombinants of SmSCR1-overexpressive and suppressed vectors. (A) Recombinant vector of pHB -SmSCR1-YFP. (B) Recombinant vector of pHB-SmSCR1-SRDX. [file Image_2.TIF]

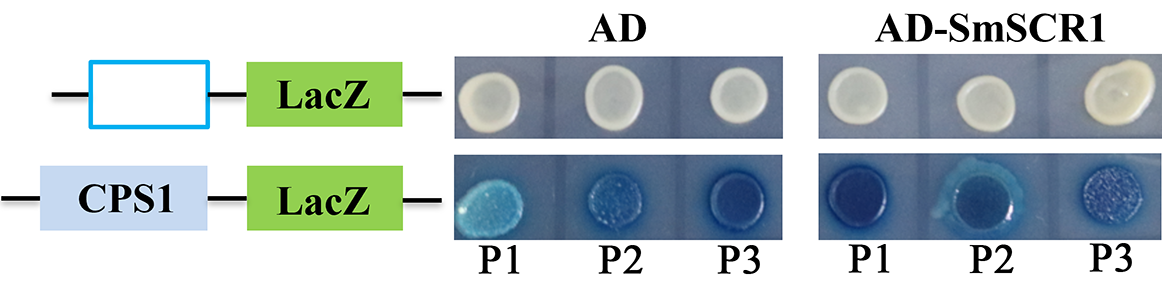

Supplement: Supplementary Figure 3 — Yeast one-hybrid assay of SmSCR1 binding to the SmCPS1 promoter. P1, P2, and P3 represent the three fragments of SmCPS1 promoter sequence from −1 to −700, −701 to −1,400, and −1,401 to −2,100 bp, relative to translation start site, respectively. [file Image_3.TIF]

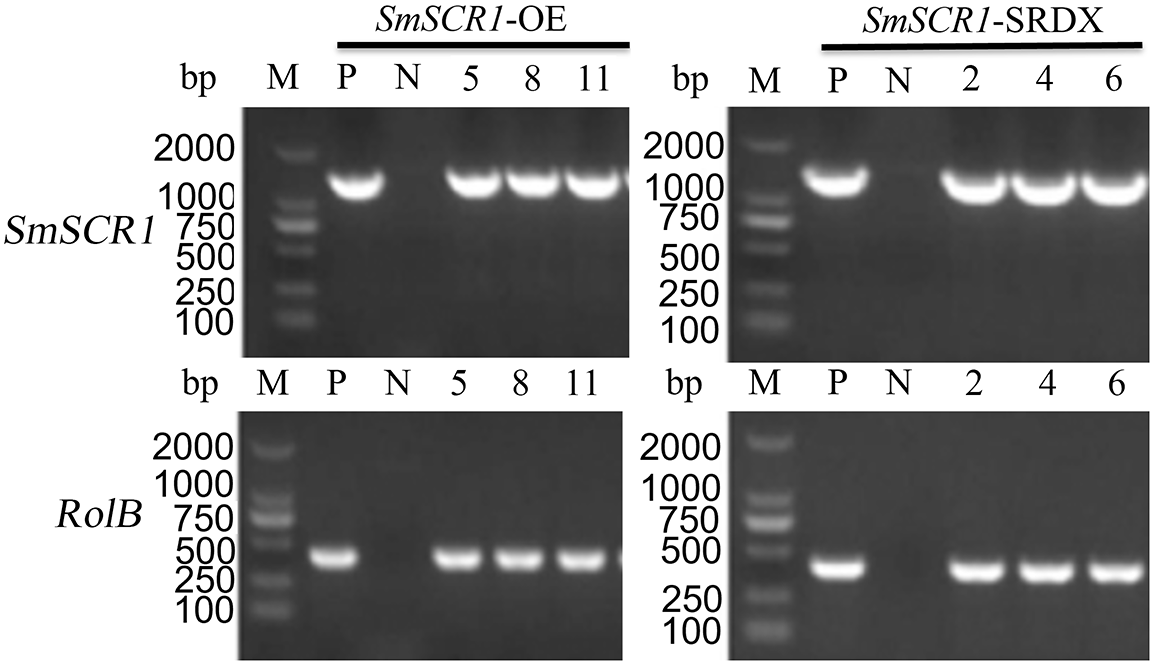

Supplement: Supplementary Figure 4 — Detection of the expression level of SmSCR1 gene in transgenic hairy root lines by qRT-PCR. [file Image_4.TIF]

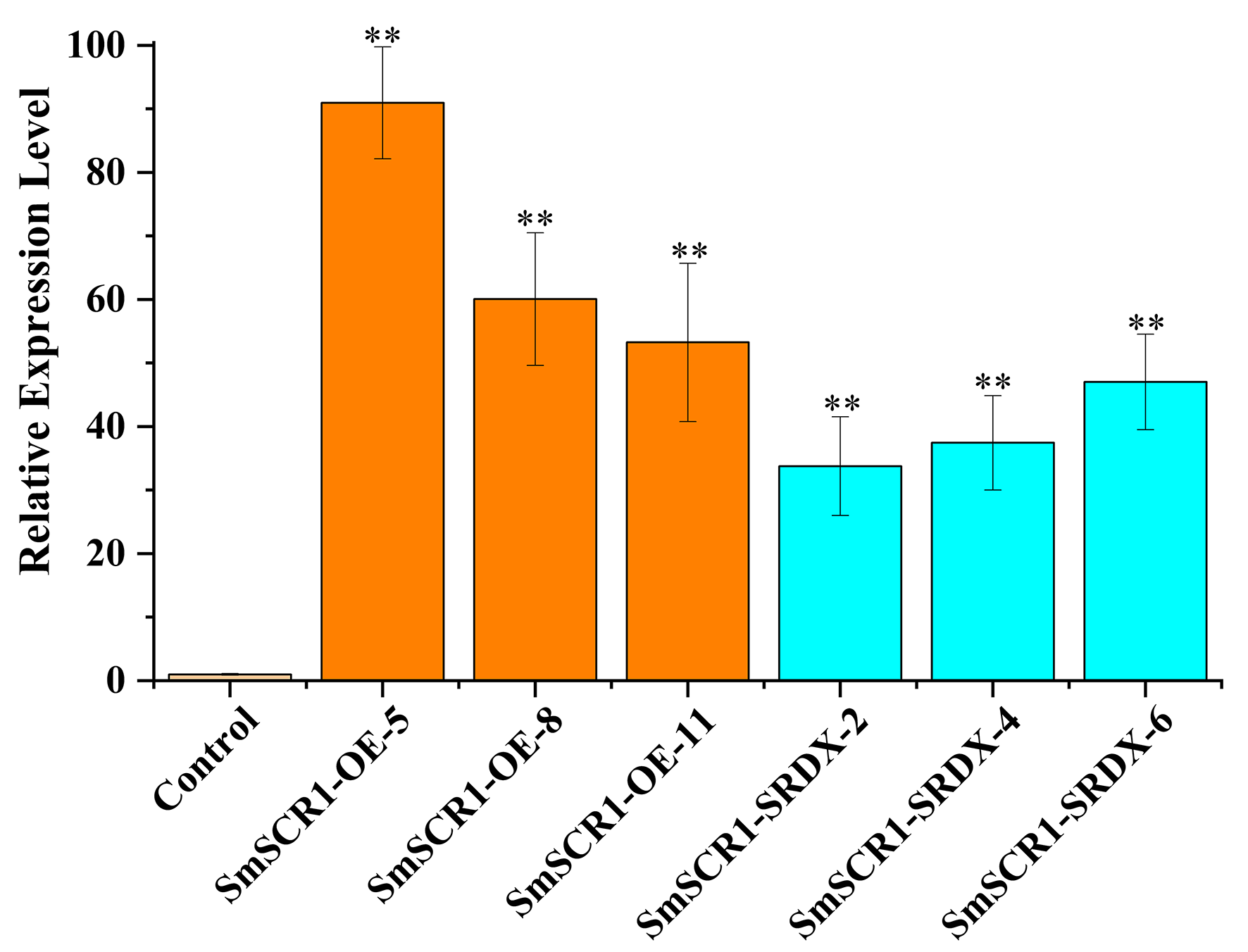

Supplement: Supplementary Figure 5 — Identification of transgenic hairy root lines by PCR. The asterisks on the bar indicate significant differences by t-test compared to the control (**P < 0.01). [file Image_5.TIF]
